# Mapping Proprioception across a 2D Horizontal Workspace

**DOI:** 10.1371/journal.pone.0011851

**Published:** 2010-07-29

**Authors:** Elizabeth T. Wilson, Jeremy Wong, Paul L. Gribble

**Affiliations:** 1 Department of Psychology, The University of Western Ontario, London, Ontario, Canada; 2 Graduate Program in Neuroscience, The University of Western Ontario, London, Ontario, Canada; 3 Department of Physiology & Pharmacology, The University of Western Ontario, London, Ontario, Canada; University of California Davis, United States of America

## Abstract

Relatively few studies have been reported that document how proprioception varies across the workspace of the human arm. Here we examined proprioceptive function across a horizontal planar workspace, using a new method that avoids active movement and interactions with other sensory modalities. We systematically mapped both proprioceptive acuity (sensitivity to hand position change) and bias (perceived location of the hand), across a horizontal-plane 2D workspace. Proprioception of both the left and right arms was tested at nine workspace locations and in 2 orthogonal directions (left-right and forwards-backwards). Subjects made repeated judgments about the position of their hand with respect to a remembered proprioceptive reference position, while grasping the handle of a robotic linkage that passively moved their hand to each judgement location. To rule out the possibility that the memory component of the proprioceptive testing procedure may have influenced our results, we repeated the procedure in a second experiment using a persistent visual reference position. Both methods resulted in qualitatively similar findings. Proprioception is not uniform across the workspace. Acuity was greater for limb configurations in which the hand was closer to the body, and was greater in a forward-backward direction than in a left-right direction. A robust difference in proprioceptive bias was observed across both experiments. At all workspace locations, the left hand was perceived to be to the left of its actual position, and the right hand was perceived to be to the right of its actual position. Finally, bias was smaller for hand positions closer to the body. The results of this study provide a systematic map of proprioceptive acuity and bias across the workspace of the limb that may be used to augment computational models of sensory-motor control, and to inform clinical assessment of sensory function in patients with sensory-motor deficits.

## Introduction

Our proprioceptive sense is important in maintaining posture and executing movement, and is based on afferents from muscle spindles, joint receptors and cutaneous receptors that signal stretch and compression of body tissue, providing information about limb position [1]. In the absence of proprioceptive feedback, deafferented patients cannot maintain the arm in a steady posture or execute controlled movements without watching their limb [2], [3]. Deafferented patients show a decreased ability to detect joint movement and an impaired ability to make accurate multi-joint movements to visual targets [4], and are unable to compensate for intersegmental interaction torques [5].

While there has been much investigation into the integration of proprioception and vision [6], [7], [8], as well as the accuracy of arm movements to both visual and proprioceptive targets [6], [9], [10], [11], [12], [13], [14], [15], [16], [17], relatively little is known about how proprioception varies across the workspace [see 18 2010 for a recent study]. Proprioception on its own is difficult to measure. Previous work investigating the psychophysics of proprioception has seldom disentangled proprioception from the various coordinate transformations implicit in the perceptual response. Some studies have required responses to visual targets [19], [20], [21], limb position matching which requires inter-hemispheric transfer of information [22], [23], [24], [25], [26], a motor response such as reaching to a target [10], [21] or reproducing a limb position [15], [22], [23], [24], [25], or some combination of the above. These factors may influence resulting estimates of proprioceptive function.

In the present paper we report experiments carried out to investigate proprioception of the passive human arm. Proprioception of the left and right arms was tested at nine workspace locations and in two directions (left-right and forwards-backwards). Proprioceptive tests required a subject to make repeated judgments about the position of their hand with respect to a remembered proprioceptive reference position. The goal of the experiment was to estimate both proprioceptive acuity (sensitivity to hand position change) and bias (perceived location of the hand), and specifically to determine whether proprioception is uniform across a 2D workspace. In a second experiment, to rule out the possibility that the small memory component of the proprioceptive testing procedure may have affected our results, we repeated the procedure using a visual reference instead of a remembered proprioceptive reference hand position.

## Methods

### Subjects

Sixty-eight healthy individuals participated in this study (aged 18 to 45 years), see Table 1 for details. Sixty-one subjects were strongly right-handed as assessed by the Dutch Handedness Questionnaire [27]. The remaining subjects were classified as neither strongly right-handed nor strongly left-handed. Their performance did not differ from that of the strongly right-handed subjects so in the analyses that follow, all subjects have been grouped together. Subjects reported no history of neurological or musculoskeletal disorder, and had normal or corrected-to-normal vision. All subjects provided written informed consent prior to participation in the study, which was approved by the University of Western Ontario Research Ethics Board.

**Table 1:** Participants.

Experiment	Total	Female	Male	Right-handed
Proprioceptive Reference	36	23	13	33
Visual Reference	36	20	16	32

Information about participants in the two experiments.

### Apparatus

**Figure 1:**
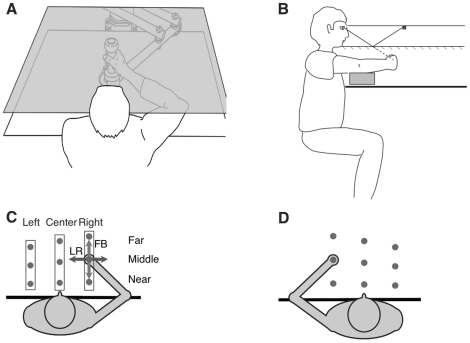
Experimental Apparatus. The subject grasped a robotic manipulandum that moved the hand along the surface of a desk. (B) Side view of set-up for Experiment 2 (visual reference experiment). (C,D) Overhead view: In Experiments 1 and 2, each participant performed proprioceptive tests at three lateral locations and along two axes (left-right (LR), forwards-backwards (FB)) either in front of the left or right shoulder or at the midline of the body. Three test positions were determined for each individual and were at 80% (far), 50% (middle) and 20% (near) of the participants maximum reach (MR). Proprioception of participants' right (C) and left (D) arms was tested.

Subjects were seated in the dark at a table adjusted to chest height. Subjects grasped the handle of an InMotion robotic linkage (In Motion Technologies, Cambridge, USA) as shown in Figure 1. An air sled was used to support the arm and allow smooth, near frictionless movement along the surface of the table (Figure 1B). The robot was programmed to move the arm from one position to another in a two-dimensional horizontal plane located just below shoulder height. A six-axis force transducer (ATI Industrial Automation, Apex, USA) inside the handle measured forces at the hand. Shoulder straps attached to the chair kept the trunk in a static position, while allowing rotation of the shoulder and elbow joints. A horizontal semi-silvered mirror was suspended 31.5 cm above the surface of the table. In Experiment 2, a red light emitting diode (LED) was suspended 12.5 cm above the semi-silvered mirror such that the reflection of the LED appeared to be in the same plane as the subject's hand (Figure 1B). Vision of the arm and the robotic manipulandum was obscured by opaque curtains in addition to the semi-silvered mirror.

### Proprioception Test Positions

Proprioceptive tests were conducted at 9 positions in the horizontal workspace, forming a 3×3 grid. The same test positions were used in Experiments 1 and 2. Each subject was randomly assigned to one of 6 groups (see Table 2): 2 (left or right arm)×3 (left, centre or right workspace). Each subject in each group completed proprioceptive tests at 3 distances from the body, located 20% (near), 50% (middle) and 80% (far) along each subject's maximum reach (Figure 1C and Figure 1D).

**Table 2:** Subject Groups.

Subject Group	Lateral Workspace Location	Arm Tested
1	Left	Left
2	Centre	Left
3	Right	Left
4	Left	Right
5	Centre	Right
6	Right	Right

Subjects were randomly assigned to one of six groups. Each group underwent proprioceptive tests at three distances from the body: 20%, 50% and 80% of their maximum reach.

Each experiment consisted of two 60-minute sessions. In the first session, subjects were tested at three distances from the body (near, middle or far) within one lateral workspace (left, centre or right). At each position proprioceptive acuity and bias was assessed using passive movements along a left-right axis (see below). In a second session on a separate day, subjects were tested at the same three positions, using passive movements along a forward-backward axis. Test direction order (left-right and forward/back) and test position order (near, middle and far) were counterbalanced across subjects.

### Test Procedure

Subjects were instructed to keep their arm muscles relaxed, and face forwards. Vision of the arm was completely blocked by opaque curtains. Each proprioceptive test consisted of 74 trials at a single test location and was performed either along a left-right or forward-backward direction.

**Figure 2:**
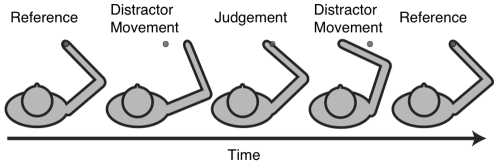
Proprioceptive Testing Procedure. On each trial, the subject's arm was moved by the robot to a reference location followed by a judgment location. Subjects made a two-alternative forced choice judgment about the position of their hand relative to the reference location. To eliminate speed or timing cues and preclude feedback about performance on a trial-to-trial basis, distractor movements were used before and after each judgement location by bringing the hand a random distance away from the test location (14±2 cm), in a random duration (700–1600 ms) and in a random direction along the test axis (left vs right or forward vs back). In Experiment 1 participants made judgments with respect to a proprioceptive reference location. In Experiment 2, participants made judgments with respect to a visual reference.

Subjects were instructed to keep their eyes closed at all times. On each trial, the subject's arm was moved to the reference position by the robotic manipulandum, and held there for 2 s (Figure 2). Next, the hand was moved away from the reference position through a distractor movement, before being brought to a judgment position where the hand was held until the subject made a two-alternative forced-choice judgement about which side along the axis of movement (left or right, forwards or backwards) the judgment position fell with respect to the reference position. The distractor movement displaced the hand 14 cm plus or minus a random distance (chosen from a gaussian with mean = 14 cm and sd = 2 cm) from the reference position along the test axis to a peripheral position before bringing the hand to a judgment position. The total duration of the distractor movement was also randomized (700–1600 ms). These distractor movements were used to eliminate any potential speed or timing cues that subject may use to judge hand position. After the subject provided a verbal response, the hand was moved through another distractor movement before being brought back to the reference position. Thus, subjects were never given direct feedback about their performance on any given trial. This was done to eliminate the possibility that subjects recalibrate their responses during testing based on direct feedback. To increase motivation throughout the experiment, subjects were given a score at 20-trial intervals that reflected their recent average performance.

Seven judgment positions were tested either along a left-right axis or a forward-backward axis: (−30, −13.3, −6.7, +0, +6.7, +13.3, +30) mm. Each judgment position was tested between 6 and 14 times (6, 12, 12, 14, 12, 12, 6). The positions furthest from the reference position were tested fewer times because subjects were expected to make essentially 100% correct judgments at these distant positions. The direction of the distractor movements (left or right; forwards or backwards) was determined pseudo-randomly such that each judgment position was approached from each of the two directions on an equal number of trials.

To familiarize the subject with the procedure, blocks of 20 practice trials were performed at the start of the experiment, until subjects demonstrated a clear understanding of the task. The majority of subjects only required a single practice block.

In Experiment 1, subjects were asked to report the current position of their hand relative to a remembered position (presented less than 2 s earlier). Importantly, this procedure avoids non-proprioceptive modalities, such as vision, motor responses and inter-hemispheric transfer of information. Intermodal performance is less accurate than intra-modal performance [7], [28], and multiple modalities introduce additional sources of error that are not easily distinguished from proprioceptive errors. By limiting our procedure to proprioceptive stimuli, we avoided such influences.

Nevertheless, one potential limiting factor of the test procedure in Experiment 1 is that at the time of response subjects were asked to recall the reference position presented earlier, and compare the current perceived position of their hand to their memory of the reference position. Error in this memory component of the task would be inseparable from error in the sense of limb position. In order to determine the potential contribution of this memory component to the results of Experiment 1, in Experiment 2 we replicated the design of the first experiment with a slightly modified proprioceptive test. In this test, subjects had to report the perceived position of their unseen hand with respect to a visual reference continuously presented on the horizontal mirror above their arm. Other than the addition of a visual reference position, all other aspects of Experiment 2 were identical to that of Experiment 1 including distractor movements and analysis. It should be noted that while the procedure used in Experiment 2 eliminates any memory component, it does involve a visual target and thus requires the transformation of visual and proprioceptive information into the same coordinate system in order to form a judgement.

In Experiment 2, the image of a red light-emitting-diode was reflected onto the semi-silvered mirror such that the visual reference appeared in the same plane as the subject's hand. To minimize the possibility of visual-proprioceptive drift [8], [11], [12], [19], [21], subjects were provided with vision of their arm for 5 seconds after every 20 trials.

**Figure 3:**
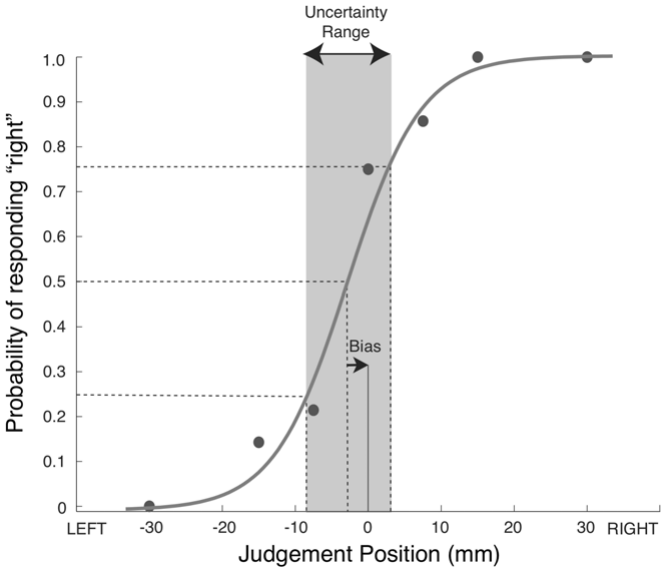
Sample Psychometric Function. At each testing location, subjects' responses were fit to a binomial model using a cumulative normal distribution function. Here we show a sample function from one subject tested at a single proprioceptive test location, along a left-right axis. Filled circles represent the proportion of times, at a given judgment location, that the subject responded that their hand was to the right of the reference location.

For each testing location, psychometric functions relating perceived hand position to actual hand position were estimated by fitting a single subject's set of responses at each judgment location to a binomial model using a cumulative normal distribution function (Figure 3). Measures of proprioceptive acuity and bias were calculated based on the psychometric function. Proprioceptive acuity was quantified as the distance along the testing axis corresponding to the middle 50th percentile range, i.e. the distance (in mm) between the 25% and 75% probabilities of reporting that their hand was to the right of the reference position (or forwards, in the case of forward-backward tests). This measure, sometimes called uncertainty range [29] provides the range over which subjects were most unsure of their hand position, and is thus inversely related to proprioceptive acuity. Uncertainty range is equivalent to ±0.674 standard deviations of the mean. Perceived hand location was quantified as the distance (in mm) between the actual hand location and the location at which the psychometric function crossed the 50% point, a measure that is sometimes called bias [29].

### Statistical Analysis

Differences in mean acuity and bias across testing locations, testing directions, and limbs were assessed using 4-factor mixed ANOVAs: distance from the body (near, middle, far; within subject) by test direction (left-right, forward-backward; within subjects) by lateral location (left, centre, right; between subjects) by limb (left, right; between subjects). Differences between individual means were assessed using Tukey post-hoc tests.

## Results

To investigate how proprioceptive acuity and bias varied across the workspace, subjects were tested at nine locations: three distances in front of their body and in three sagittal axes and in two directions. Differences in proprioception for the right and left arms were tested between subjects. In the figures that follow, we show the results of Experiment 1 (in which a proprioceptive reference position was used) on the left side, and the results of Experiment 2 (in which a visual reference was used) on the right.

### Proprioceptive Acuity

**Figure 4:**
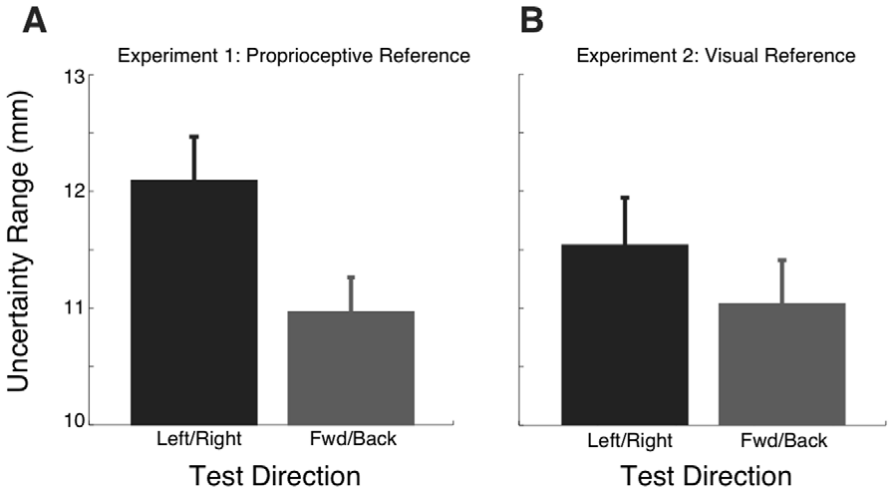
Proprioceptive Acuity as a function of testing direction. The width of the uncertainty range is shown as a function of testing direction, left-right (LR) vs forward-backward (FB), for Experiment 1 (A) and Experiment 2 (B). Data shown are averaged over workspace position and limb.

Tables 3 and 4 show mean acuity as a function of testing location, testing direction, and limb, for both Experiments 1 and 2. We observed a significant effect of test direction (left-right vs forward-backward) on uncertainty range (Experiment 1: p<0.05, Experiment 2: p<0.001, Figure 4). Subjects were significantly more sensitive (smaller uncertainty range) in the forward-backward axis than along the left-right direction.

**Table 3:** Experiment 1: Mean proprioceptive acuity.

Left/Right Axis												
	Left Hand						Right Hand					
	Left		Centre		Right		Left		Centre		Right	
Far	12.99	−4.12	15.58	−11.42	12.26	−4.20	12.82	−3.28	10.97	−2.96	12.10	−2.44
Mid	11.98	−4.07	14.83	−3.94	11.70	−6.62	13.75	−6.35	11.37	−3.95	11.54	−1.86
Near	10.50	−3.38	13.75	−4.04	9.42	−3.03	11.28	−2.04	10.13	−4.68	11.70	−2.71

Forward/Backward Axis												
Far	11.14	−3.00	11.42	−3.57	10.18	−4.14	12.40	−4.26	11.06	−2.37	8.50	−1.68
Mid	10.84	−3.14	11.28	−2.73	10.79	−2.01	11.29	−2.73	11.64	−1.52	11.00	−1.14
Near	10.54	−3.50	12.55	−4.40	9.37	−2.86	10.70	−3.44	11.11	−3.84	10.25	−1.16

Mean (± 1 standard deviation) proprioceptive acuity (mm) in Experiment 1 as a function of testing location, testing direction and limb.

**Table 4:** Experiment 2: Mean proprioceptive acuity.

Left/Right Axis												
	Left Hand						Right Hand					
	Left		Centre		Right		Left		Centre		Right	
Far	11.29	−2.53	12.74	−3.15	9.68	−2.23	13.60	−2.78	10.48	−2.76	14.74	−4.93
Mid	11.56	−4.37	17.02	−5.75	7.59	−4.69	9.88	−2.18	11.84	−2.95	13.05	−3.73
Near	10.93	−2.88	10.54	−2.33	10.65	−4.10	9.34	−1.98	7.27	−2.35	9.50	−2.02

Forward/Backward Axis												
Far	9.70	−3.27	16.07	−4.11	10.14	−3.31	12.86	−2.45	9.15	−2.14	11.47	−5.82
Mid	9.62	−2.40	11.38	−2.61	10.84	−3.16	12.17	−4.71	11.46	−3.18	12.37	−5.24
Near	7.19	−3.98	9.24	−2.69	10.93	−5.02	10.75	−2.28	9.08	−1.32	10.60	−2.29

Mean (± 1 standard deviation) proprioceptive acuity (mm) in Experiment 2 as a function of testing location, testing direction and limb.

**Figure 5:**
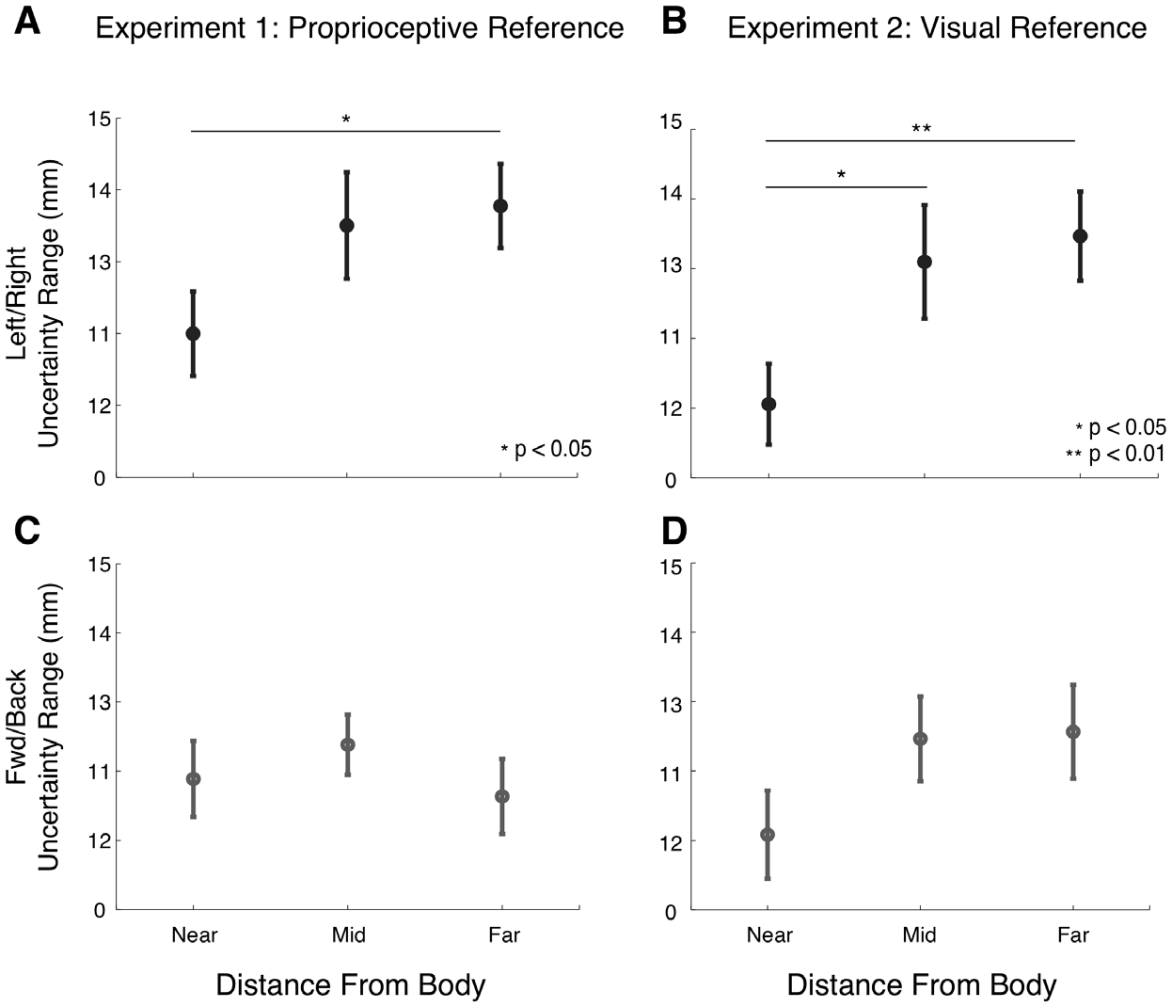
Proprioceptive Acuity as a function of distance from the body. Uncertainty range is shown as a function of distance from the body (near, middle, far) for left-right test directions (A,B) and forward-backward directions (C,D) in Experiments 1 (A,C) and 2 (B,D). Data shown are averaged over workspace position and limb.

We also observed a significant effect of distance from the body on proprioceptive acuity, but only for tests in the left-right direction. Figure 5 gives observed differences in acuity for hand locations at the different distances from the body. In both Experiments 1 and 2, for tests along a left-right axis, subjects were more sensitive at the near position than at the far position (Experiment 1: p<0.05, Experiment 2: p<0.01, Tukey). In Experiment 2 we also observed a significant difference between near and middle positions (p<0.05).

**Figure 6:**
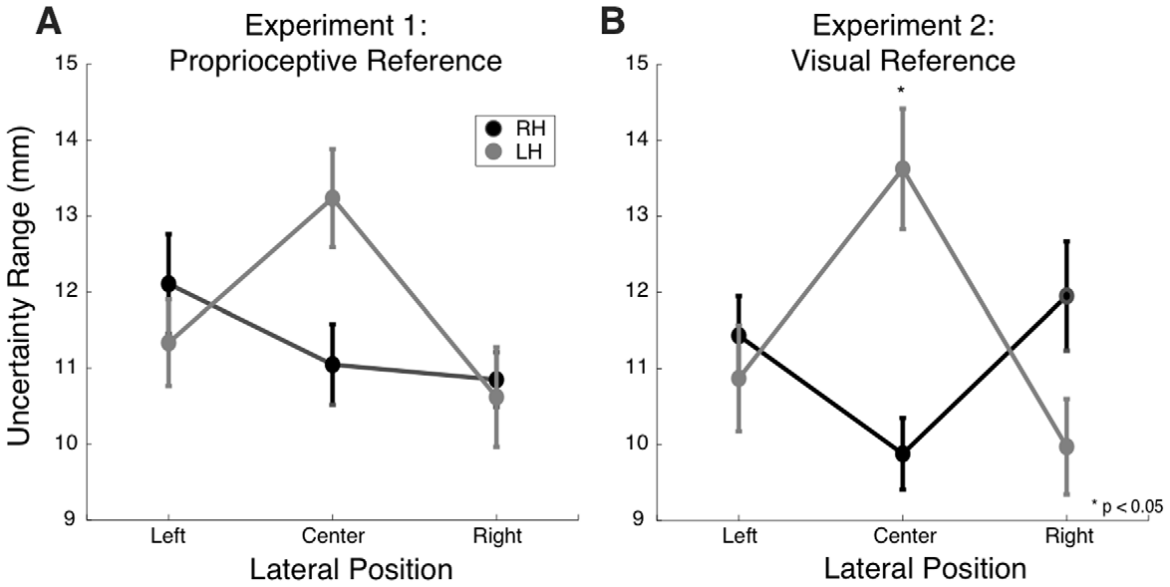
Proprioceptive Acuity as a function of lateral position. Uncertainty range is shown as a function of lateral position (left, centre, right) for left and right arms, in Experiments 1 (A) and 2 (B). Data shown are averaged over distance from the body and test direction.

In Experiment 2, a significant interaction effect was observed between limb (left, right) and lateral position (p<0.05, ANOVA, see Figure 6B). At the centre of the workspace, the right hand was more sensitive than the left (p<0.05). No statistically reliable differences were observed between limbs on the right (p = 0.65) or left (p = 1) workspace locations. The interaction between limb and lateral position was not statistically reliable in Experiment 1 (p = 0.188), although a similar pattern is clearly apparent (Figure 6A).

We observed no other statistically reliable differences in proprioceptive acuity.

### Proprioceptive Bias

Proprioceptive bias reflects the shift in the perceived position of the hand relative to the its actual position. In the left-right test direction, a positive bias indicates that the subject perceived their hand to be to the right of its physical position, while in the forward-backward test direction, a positive bias indicates that the hand was perceived to be further away (forwards) from the body than its actual position. Tables 5 and 6 show mean acuity as a function of testing location, testing direction, and limb, for both Experiments 1 and 2.

**Table 5:** Experiment 1: Mean proprioceptive bias.

Left/Right Axis												
	Left Hand						Right Hand					
	Left		Mid		Right		Left		Mid		Right	
Far	−2.91	−1.89	−5.42	−3.08	−3.45	−2.39	2.07	−3.62	2.07	−2.63	2.03	−3.36
Mid	−2.65	−1.80	−4.85	−2.74	−3.24	−1.42	1.97	−3.86	2.17	−2.07	2.91	−3.32
Near	−1.97	−2.59	−4.24	−2.70	−1.99	−1.05	0.71	−2.10	0.49	−1.33	1.99	−2.43

Forward/Backward Axis												
Far	1.65	−3.52	−1.27	−5.10	1.19	−3.39	2.78	−1.18	1.02	−3.08	1.57	−2.21
Mid	2.12	−3.10	0.07	−5.74	0.96	−2.58	0.84	−1.84	1.13	−1.91	0.67	−1.75
Near	−0.85	−3.62	−0.09	−5.61	1.24	−1.63	1.09	−3.42	−0.06	−3.03	−2.15	−1.42

Mean (± 1 standard deviation) proprioceptive bias in Experiment 1 as a function of testing location, testing direction and limb.

**Table 6:** Experiment 2: Mean proprioceptive bias.

Left/Right Axis												
	Left Hand						Right Hand					
	Left		Mid		Right		Left		Mid		Right	
Far	−2.87	−10.09	−8.12	−3.51	−2.57	−2.14	6.22	−4.16	4.07	−6.69	6.60	−2.72
Mid	−6.12	−10.27	−10.42	−4.59	−2.79	−2.10	6.02	−3.79	3.77	−3.03	4.62	−3.63
Near	−4.39	−5.98	−5.22	−2.03	−3.69	−3.88	1.45	−3.01	3.75	−2.86	3.65	−2.04

Forward/Backward Axis												
Far	−2.39	−8.59	1.52	−6.87	3.40	−3.92	1.99	−5.77	−2.45	−4.36	−2.11	−4.03
Mid	−5.02	−3.48	−0.06	−4.70	1.74	−4.22	3.39	−3.83	−0.08	−5.30	0.55	−2.59
Near	−5.46	−2.39	1.19	−6.69	4.03	−4.94	1.61	−5.37	0.82	−5.55	−2.47	−4.34

Mean (± 1 standard deviation) proprioceptive bias in Experiment 2 as a function of testing location, testing direction and limb.

**Figure 7:**
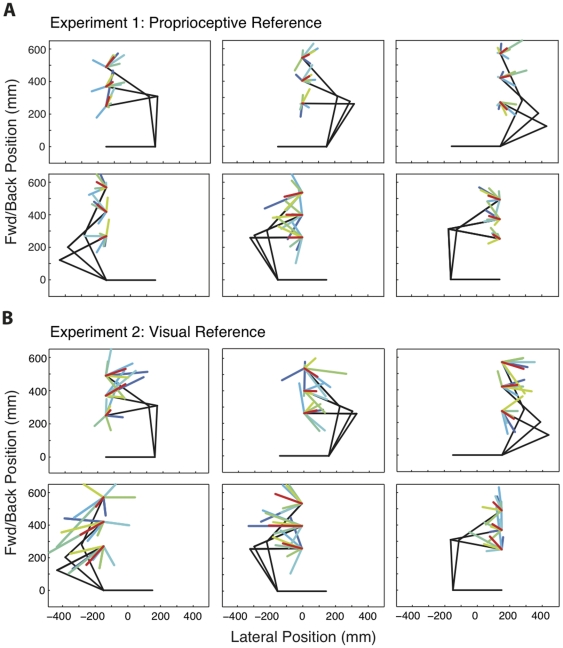
Proprioceptive Bias across the workspace. Proprioceptive bias is plotted as a vector (the vector sum of bias in the left-right and forward-backward directions) for individual subjects (gray) and the group mean (black), for the left and right hands at three lateral positions and three distances from the body, for Experiment 1 (A) and 2 (B). For purposes of visualization, vector length has been increased by a factor of 20.

In both Experiments 1 and 2, we observed a significant main effect of limb on proprioceptive bias in the left-right direction. At all distances from the body and at all lateral locations, estimates of the location of the right hand were biased toward the right, and those of the left hand were biased toward the left (p<0.01 in all cases). Thus the right hand was perceived to be right of veridical, and the left hand was perceived to be left of its actual position. Figure 7 gives bias, plotted as vectors (the vector sum of left-right and forward-backward biases) across workspace positions for both the right and left arms. Vectors for individual subjects (gray) and group results (black) are shown.

**Figure 8:**
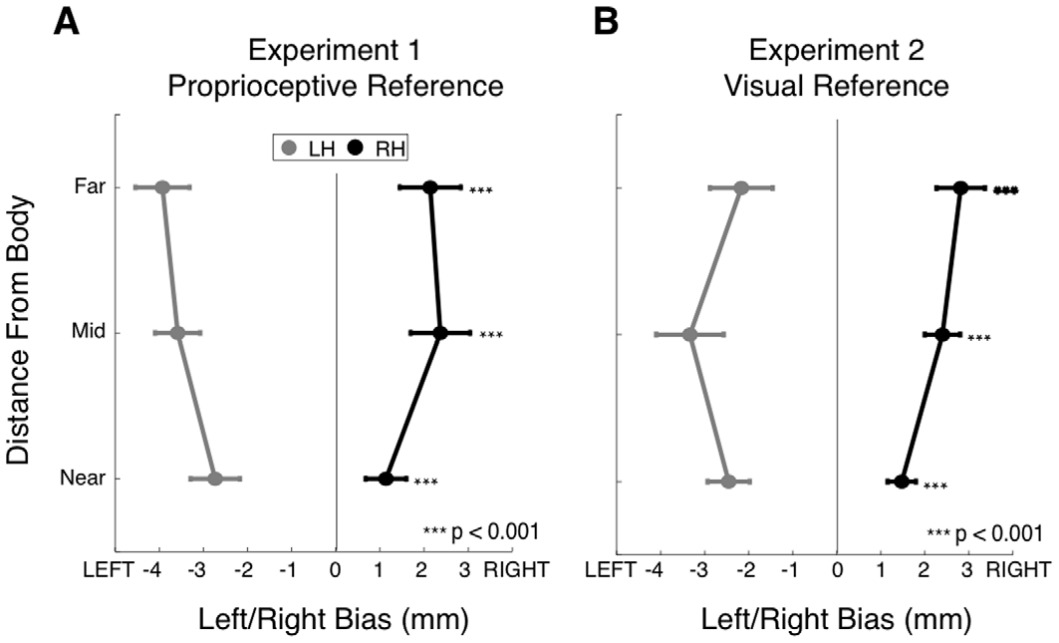
Proprioceptive Bias as a function of limb and distance from the body. Proprioceptive bias in the left-right direction is shown as a function of distance from the body (near, middle, far) for the left and right arms, in Experiments 1 (A) and 2 (B). Data shown are averaged over lateral position.

In both Experiment 1 and 2, there was a significant interaction effect of distance from the body (near, middle, far) and limb (left, right) on bias, when tested in the left-right direction (p<0.05). We observed a trend whereby bias was smaller for hand positions closer to the body, and greater for hand positions farther from the body (Figure 8A, B).

**Figure 9:**
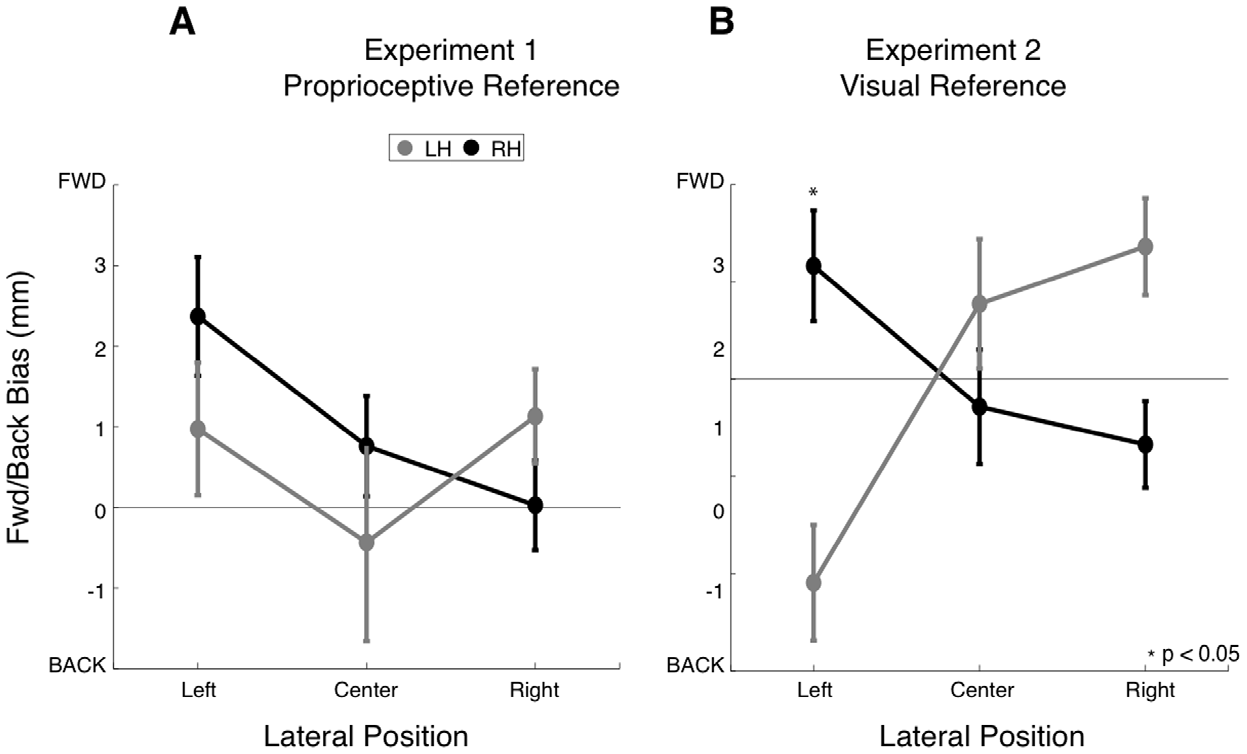
Proprioceptive Bias as a function of lateral position. Proprioceptive bias along the forward-backward direction is shown as a function of lateral position (left, centre, right) for the left and right hands, in Experiment 1 (A) and 2 (B). Data shown are averaged over distance from body.

In Experiment 2, we observed a reliable interaction effect between limb and lateral position on proprioceptive bias in the forward-backward direction (p<0.01, Figure 9B). When tested in the left side of the workspace, the left hand was perceived to be closer to the body (negative values of forward-backward bias) than the actual hand position and the right hand was perceived to be further away from the body. There was a trend towards the opposite effect on the right side of the workspace. Estimates of the position of the right hand were biased towards the body and for the left hand were biased away from the body. When tested at the midline of the body, biases in the perceived positions of the two hands did not differ reliably (p = 0.92). This interaction was not seen in Experiment 1 (p = 0.50, Figure 9A).

No other statistically reliable differences in proprioceptive bias were observed.

### Control Tests

Without visual feedback, estimations of limb position tend to drift [8], [11], [12], [21]. These studies have shown that the perceived position of the right hand tends to drift towards the body over time and to the right. Drift has been attributed to a misalignment between the proprioceptive and visual systems. Drift is halted when vision of the limb is provided [21], when passive or active movements [30], or when isometric contractions are performed with the target limb [21]. Previous results suggest that drift does not occur within the proprioceptive modality over short time periods. Desmurget et al. [19] showed that drift did not occur over two reaches in a 20 s period in a hand position matching task, and Chapman et al. [14] found that subjects could accurately point to kinesthetically presented targets after a 2 s delay. Considering these findings, we are confident that drift was not influencing the results of Experiment 1, where the reference position was presented via proprioception and the time elapsed between presentation of the reference position and the judgment position was less than two seconds.

**Figure 10:**
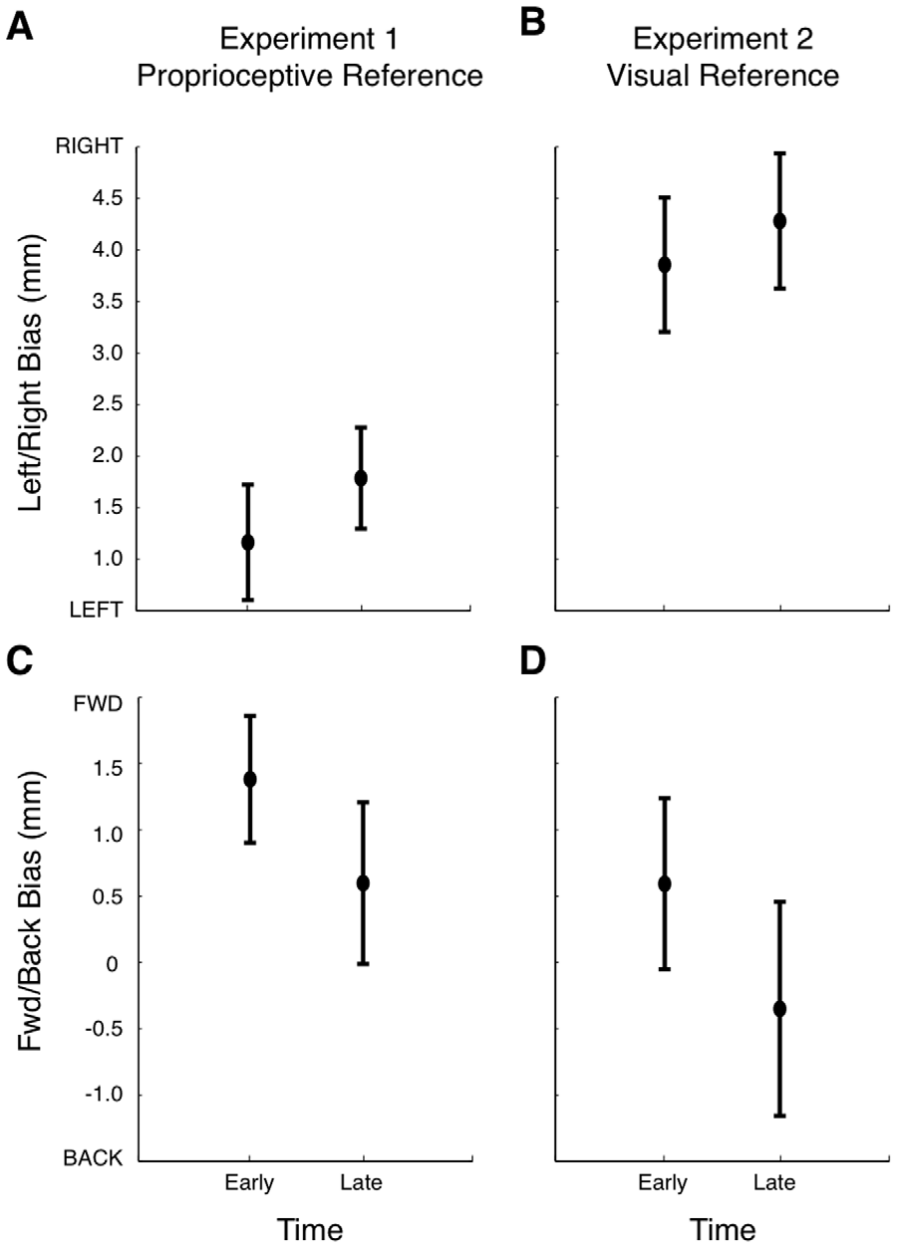
Tests of proprioceptive drift of the right arm. Change in proprioceptive bias (mean ± 1 standard error) over time for early trials (first 7 trials of each block) and late trials (last 7 trials of each block) in the left-right (A,B) and forward-backward (C,D) test directions for Experiment 1 (A,C) and 2 (B,D). Data shown are averaged over workspace position.

**Figure 11:**
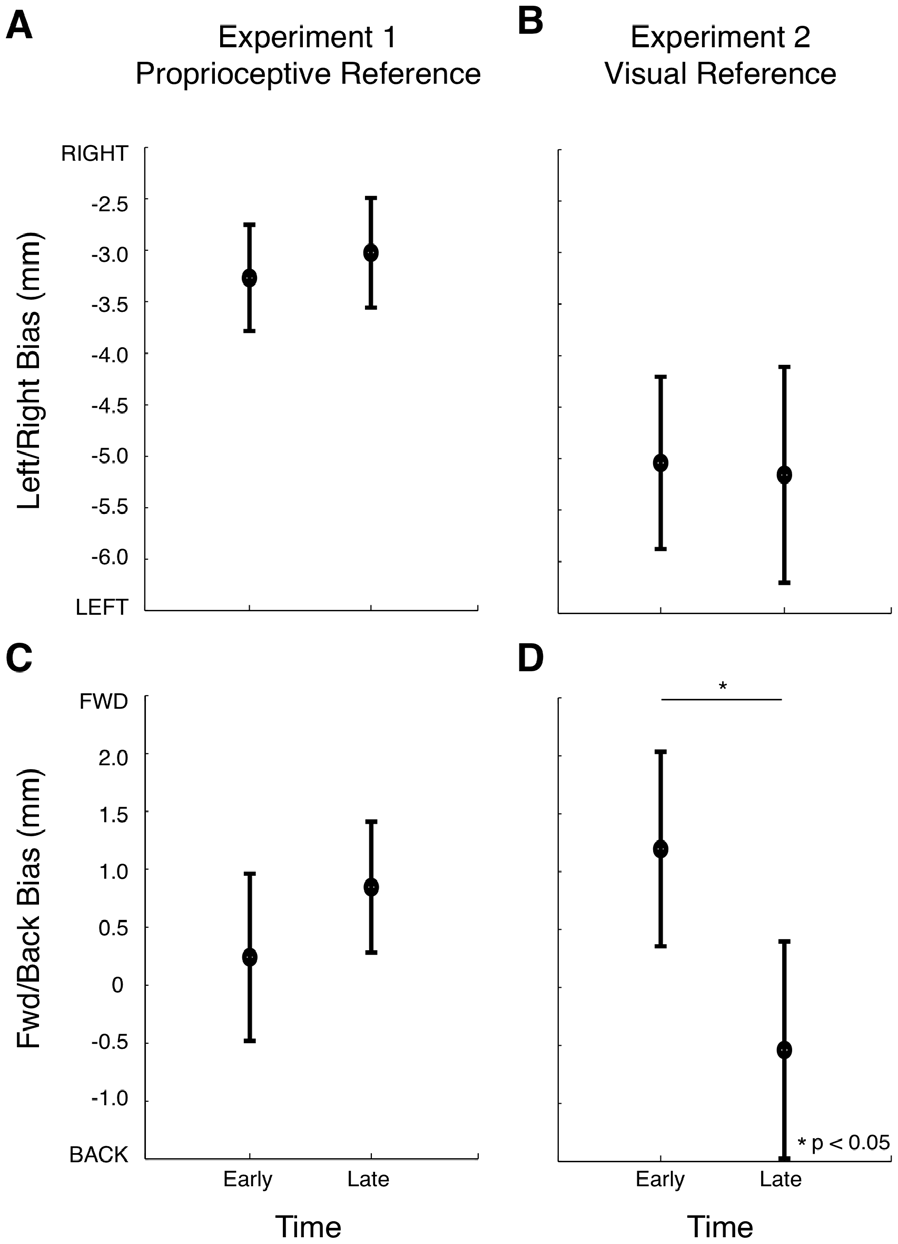
Tests of proprioceptive drift of the left arm. Change in proprioceptive bias (mean ± 1 standard error) over time for early trials (first 7 trials of each block) and late trials (last 7 trials of each block) in the left-right (A,B) and forward-backward (C,D) test directions for Experiment 1 (A,C) and 2 (B,D). Data shown are averaged over workspace position.

Nevertheless, we tested whether drift was occurring by comparing the first third of the block (the first 7 trials) with the last third of the block (the last 7 trials) of each 20-trial block, for both Experiments 1 and 2. Completion of a block took approximately 2.5 min. In Experiment 1, no change in proprioceptive bias was observed over time either in the left-right or forward-backward directions for either limb (p>0.5 in both cases, see Figure 10A,C and Figure 11A,C). In Experiment 2, subjects made judgments about the perceived position of their hand with respect to a visual reference. Vision of the hand was provided at the beginning of every 20-trial block. There was no change in left-right bias over time for either limb (Figure 10B, Figure 11B), nor in the forward-backward direction for the right limb (Figure 10D). However for the left limb, in the forward-backward direction, bias was slightly further towards the body (more negative values of bias) later in the block (Figure 11D). This suggests that a small drift may have occurred between the vision of the LED and the perceived position of the hand, in a direction consistent with previous findings [12], [21].

It is possible that judgements about limb position could be influenced if subjects actively resisted the movements imposed by the robot arm, and/or actively pushed against the handle during the judgment phase of each trial. In order to assess this possibility, we examined the forces being externally imposed on the robot handle during the judgement phase of each trial. There were no statistically reliable differences in mean force across test conditions (p>0.5 in all cases), suggesting that subjects were not actively pushing against the handle, or resisting the movements imposed by the robot.

## Discussion

This study examined proprioception of the human arm using a novel proprioceptive testing procedure. Two experiments are reported that assessed whether differences in proprioceptive acuity or bias exist across a horizontal workspace or between the right and left limbs. We found that proprioceptive acuity is not uniform across the workspace. Acuity is greater for hand positions closer to the body, and is greater in a forward-backward direction than in a left-right direction. In addition, at the midline of the body, acuity for the right arm is greater than for the left. Differences in proprioceptive bias were also observed. There was a robust difference in proprioceptive bias along a lateral axis, between the two limbs. The right hand was perceived to be to the right of its actual position, while the left hand was perceived to be shifted to the left. In addition bias tended to be smaller for hand positions closer to the body.

### Proprioceptive Acuity

In our study, subjects demonstrated better proprioceptive acuity for hand positions close to the body. This pattern is consistent with a previous study that compared proprioceptive localization at three positions in a horizontal workspace [10]. The authors report that subjects were more precise at positions closer to the shoulder of the investigated arm. This pattern was not seen in a recent investigation into proprioception at the joint-level [31], in which distance from fingertip to shoulder was not predictive of the accuracy of elbow angle estimation.

We also found that proprioceptive acuity is greater in the forward-backward test direction than in the left-right test direction. Again, this is similar to previous work by van Beers et al. [10] who found that localization of the hand was more precise in the direction radial to the shoulder compared to the azimuthal direction.

Both of these findings are consistent with the idea that differences in proprioceptive acuity are related to limb geometry [10], [32], [33], [34]. For a given relative positional change at the hand (e.g. a 10 mm movement to the right), different limb configurations result in different relative changes in joint angle. Since stretch of muscle spindles is directly related to joint angle change, we might expect to find measurable differences in perceptual thresholds at different positions in the workspace and for different movement directions. Given proprioceptive acuity at the joints (shoulder and elbow), we can then predict proprioceptive acuity, at a workspace location and/or in a specific test direction, based on the resulting changes in joint angle for a displacement of the hand of a given magnitude. Larger total changes in joint angle ought to be better discriminated and therefore provide higher proprioceptive acuity.

We estimated changes in joint angle for a typical subject (distance from body midline to shoulder = 15 cm, length of upper arm = 31 cm, length of lower arm = 32 cm). Collapsed across workspace locations, mean total change in joint angle (elbow plus shoulder) is greater in the forward-backward test direction than in the left-right test direction (FB: 22.4, LR: 16.3). Thus, forward-backward acuity is predicted to be greater than left-right acuity, which is in agreement with our empirical findings. This hypothesis also suggests greater acuity due to larger joint angular changes associated with hand movements in the left-right direction closer to the body (near: mean = 18.0, mid: 15.1, far: 16.0). Again, this pattern was seen in our observed results.

In the present study, the only limb-dependent difference in acuity was seen at the midline of the body. Surprisingly, here we found that acuity of the right arm was better than that of the left. This is contrary to what we expected based on previous studies [23], [24], [25], [35]. However, other studies have failed to demonstrate any limb-dependent differences in proprioception [13], [36], although no studies to our knowledge have shown better acuity for the right arm. Presumably these heterogeneous findings in the literature relate to differences in experimental procedure. In previous investigations by Goble and colleagues [23], [24], [25] the workspace tested was not the same as in the current study, in which we tested a more central workspace region that was shared between the two limbs. It has also been shown that asymmetries in limb proprioception are more pronounced for larger amplitude movements [25]. The current experiment tested relatively small amplitude joint changes. Previous studies that suggest a left arm and right hemisphere advantage for proprioception involved active movement [23], [24], [25], [35], while the current study investigated perception of the passive limb.

The measures of perceptual acuity reported in the current study are smaller in magnitude of those reported in a recent study of proprioception at the elbow [31] (the mean precision reported in [31] was converted to uncertainty range at the hand, resulting in a value of 44.8 mm, compared to the grand mean from the current study for the right limb of 11.09 mm). It should be noted that the procedures used in the two studies differed considerably. In the Fuentes and Bastian study, subjects estimated the position of their unseen right limb with a visual cursor controlled by a joystick in their left hand. In addition, Fuentes and Bastian investigated errors in the estimation of elbow angle, while shoulder angle was fixed.

### Proprioceptive Bias

In both Experiments 1 and 2, subjects showed a robust limb-dependent difference in proprioceptive bias in the left-right direction. Subjects reported that the position of their right hand was rightward of the reference position, and that their left hand was to the left of the reference position. It should be noted that these psychophysical biases are relative to a reference position (in Experiment 1, a remembered proprioceptive position and in Experiment 2 a continuous visual position). This is a feature common to other psychophysical measurements of proprioception that involve a comparison between sensed limb position and any other reference, be it a remembered location, visual reference or the other limb. In this study, the reference position was presented to subjects using different modalities in the two experiments. Since both experiments show the same hand-dependent biases, it is likely that this pattern is at least in part due to biases in the perceived position of the limb at the judgment position, and not simply an error in recalling the reference position.

Vindras et al. [17] and Desmurget et al. [19] report a similar shift in the perceived position of the right hand. In both studies, subjects used a laser spot controlled by a joystick in the left hand to localize the unseen right hand on the left and right sides of the workspace (26 cm from sternum and 12 cm to left and right of midline). At both positions subjects indicated the position of the right hand to be further to the right than the actual hand position. Vindras et al. [17] also offer evidence that pointing errors are correlated with biases in the perception of initial hand position. Contrary to suggestions by Dijkerman and de Haan [37], this suggests that biases in the perception of limb position affect movement.

The measures of perceptual bias of this study fall within the range reported in Fuentes and Bastian [31] (accuracy in the passive elbow angle task was estimated from Figure 3B and converted to bias in mm at the hand, resulting in a range from −72.3 mm to 56.9 mm). The authors observed biases similar to those in the current study at elbow angles or 45 and 60 degrees; however the biases observed at more extreme elbow angles were considerably larger than those seen at similar limb configurations in the current study (see above for differences in the experimental procedures between the two studies). The origin of the observed patterns of proprioceptive bias are unknown. One possibility is that patterns of muscle spindle preferred directions [38], [39], [40] or their relation to muscle and limb geometry [41] may result in biases in perceived hand locations. Another possibility is that proprioceptive function may be influenced by the natural statistics of action, that is, the frequency that certain limb configurations (e.g. those corresponding to hand positions close to the body) occur in daily life [42], [43].

Our findings offer a new interpretation of previous proprioceptive phenomena, such as the “overlap effect” [10], [44], [45]. When subjects are instructed to point to their unseen hand with the opposite limb, their responses are biased such that the right hand is left of the left hand, i.e. the hands overlap. This tendency is referred to as the overlap effect. The pattern of proprioceptive biases reported in the current study offer an explanation of this phenomenon. Perception of the right hand's position is biased to the right, and perception of the left hand's position is biased to the left. When subjects are asked to align their two hands in the absence of vision, they are aligning the perceived positions of their hands. The resulting actual hand positions are overlapped due to the biases between the perceived and actual positions of the hands.

The pattern of proprioceptive biases reported here may provide a functional advantage in performing bimanual tasks. The majority of our object manipulations and bimanual interactions are performed in the centre of the workspace [42]. When reaching for an object or performing a bimanual task, it may be advantageous to overshoot with each limb, and thus overlap the target, and rely on tactile feedback to halt the movement, rather than undershoot the target and execute additional corrective movements.

Although the results of Experiments 1 and 2 were qualitatively very similar, one difference was quite apparent: larger biases were observed in the visual experiment (Figure 7). One explanation for this difference is that parallax made visual localization of the reference difficult. At the beginning of each block, the room was illuminated and subjects aligned their hand with the reflection of the LED. The visual information regarding the surrounding environment provided a reference frame from which the depth of the reflection could be judged. During the testing, the room was dark, except for the LED, making it more difficult for subjects to judge the depth of the reflected visual target. The lack of depth cues may have resulted in systematic mis-localization of the visual reference position, and thus may have resulted in larger biases in Experiment 2.

### Conclusion

The findings reported here represent a systematic mapping of proprioceptive function across space and between the two limbs, and indicate that proprioceptive acuity and bias are not uniform across the workspace. Differences in proprioceptive function may be related, in part, to limb geometry and differences in the relative stretch of muscles as the limb configuration changes. The natural statistics of action, that is, biases in the frequency of certain limb positions [42], [43] or spatial biases in muscle spindle firing rates [38], [39], [40], [41] may also play a role in determining differences in proprioceptive function over the workspace. The results presented here will benefit those developing computational models of sensory-motor control, by allowing the incorporation of more accurate models of proprioceptive function. Our findings may also be used to inform clinical assessment of sensory function in patients with sensory-motor deficits.

## References

[1] McCloskey DI (1978). Kinesthetic sensibility.. Physiol Rev.

[2] Rothwell JC, Traub MM, Day BL, Obeso JA, Thomas PK (1982). Manual motor performance in a deafferented man.. Brain.

[3] Sanes JN, Mauritz KH, Evarts EV, Dalakas MC, Chu A (1984). Motor deficits in patients with large-fiber sensory neuropathy.. Proc Natl Acad Sci U S A.

[4] Gordon J, Ghilardi MF, Ghez C (1995). Impairments of reaching movements in patients without proprioception. I. Spatial errors.. J Neurophysiol.

[5] Sainburg RL, Ghilardi MF, Poizner H, Ghez C (1995). Control of limb dynamics in normal subjects and patients without proprioception.. J Neurophysiol.

[6] van Beers RJ, Sittig AC, Denier van der Gon JJ (1996). How humans combine simultaneous proprioceptive and visual position information.. Exp Brain Res.

[7] Darling WG, Miller GF (1993). Transformations between visual and kinesthetic coordinate systems in reaches to remembered object locations and orientations.. Exp Brain Res.

[8] Smeets JBJ, van den Dobbelsteen JJ, de Grave DDJ, van Beers RJ, Brenner E (2006). Sensory integration does not lead to sensory calibration.. Proc Natl Acad Sci U S A.

[9] Adamovich SV, Berkinblit MB, Fookson O, Poizner H (1998). Pointing in 3D space to remembered targets. I. Kinesthetic versus visual target presentation.. J Neurophysiol.

[10] van Beers RJ, Sittig AC, Denier van der Gon JJ (1998). The precision of proprioceptive position sense.. Exp Brain Res.

[11] Brown LE, Rosenbaum DA, Sainburg RL (2003). Movement speed effects on limb position drift.. Exp Brain Res.

[12] Brown LE, Rosenbaum DA, Sainburg RL (2003). Limb position drift: implications for control of posture and movement.. J Neurophysiol.

[13] Carson RG, Elliott D, Goodman D, Dickinson J (1990). Manual asymmetries in the reproduction of a 3-dimensional spatial location.. Neuropsychologia.

[14] Chapman CD, Heath MD, Westwood DA, Roy EA (2001). Memory for kinesthetically defined target location: evidence for manual asymmetries.. Brain Cogn.

[15] Chieffi S, Conson M, Carlomagno S (2004). Movement velocity effects on kinaesthetic localisation of spatial positions.. Exp Brain Res.

[16] Chokron S, Colliot P, Atzeni T, Bartolomeo P, Ohlmann To (2004). Active versus passive proprioceptive straight-ahead pointing in normal subjects.. Brain Cogn.

[17] Vindras P, Desmurget M, Prablanc C, Viviani P (1998). Pointing errors reflect biases in the perception of the initial hand position.. J Neurophysiol.

[18] Fuentes CT, Bastian AJ Where is your arm? Variations in proprioception across space and tasks.. J Neurophysiol.

[19] Desmurget M, Vindras P, Gr√©a H, Viviani P, Grafton ST (2000). Proprioception does not quickly drift during visual occlusion.. Exp Brain Res.

[20] Sittig AC, Denier van der Gon JJ, Gielen CC (1985). Separate control of arm position and velocity demonstrated by vibration of muscle tendon in man.. Exp Brain Res.

[21] Wann JP, Ibrahim SF (1992). Does limb proprioception drift?. Exp Brain Res.

[22] Adamo DE, Martin BJ (2009). Position sense asymmetry.. Exp Brain Res.

[23] Goble DJ, Brown SH (2008). Upper limb asymmetries in the matching of proprioceptive versus visual targets.. J Neurophysiol.

[24] Goble DJ, Brown SH (2007). Task-dependent asymmetries in the utilization of proprioceptive feedback for goal-directed movement.. Exp Brain Res.

[25] Goble DJ, Lewis CA, Brown SH (2006). Upper limb asymmetries in the utilization of proprioceptive feedback.. Exp Brain Res.

[26] Darling WG (1991). Perception of forearm angles in 3-dimensional space.. Exp Brain Res.

[27] Van Strien JW (1992). Classification of left- and right-handed research participants.. Ned Tijdschr Psychol.

[28] Laufer Y, Hocherman S (1998). Visual and kinesthetic control of goal-directed movements to visually and kinesthetically presented targets.. Percept Mot Skills.

[29] Henriques DYP, Soechting JF (2003). Bias and sensitivity in the haptic perception of geometry.. Exp Brain Res.

[30] Paillard J, Brouchon M (1968).

[31] Fuentes CT, Bastian AJ (2010). Where is your arm? Variations in proprioception across space and tasks.. J Neurophysiol.

[32] Rossetti Y, Meckler C, Prablanc C (1994). Is there an optimal arm posture? Deterioration of finger localization precision and comfort sensation in extreme arm-joint postures.. Exp Brain Res.

[33] Scott SH, Loeb GE (1994). The computation of position sense from spindles in mono- and multiarticular muscles.. J Neurosci.

[34] Hall LA, McCloskey DI (1983). Detections of movements imposed on finger, elbow and shoulder joints.. J Physiol.

[35] Sainburg RL (2002). Evidence for a dynamic-dominance hypothesis of handedness.. Exp Brain Res.

[36] Wrisberg CA, Winter TP (1985). Reproducing the end location of a positioning movement: the long and short of it.. J Mot Behav.

[37] Dijkerman HC, de Haan EHF (2007). Somatosensory processes subserving perception and action.. Behav Brain Sci.

[38] Jones KE, Wessberg J, Vallbo AB (2001). Directional tuning of human forearm muscle afferents during voluntary wrist movements.. J Physiol.

[39] Bergenheim M, Ribot-Ciscar E, Roll JP (2000). Proprioceptive population coding of two-dimensional limb movements in humans: I. Muscle spindle feedback during spatially oriented movements.. Exp Brain Res.

[40] Roll JP, Bergenheim M, Ribot-Ciscar E (2000). Proprioceptive population coding of two-dimensional limb movements in humans: II. Muscle-spindle feedback during “drawing-like” movements.. Exp Brain Res.

[41] Herrmann U, Flanders M (1998). Directional tuning of single motor units.. J Neurosci.

[42] Howard IS, Ingram JN, K√∂rding KP, Wolpert DM (2009). Statistics of natural movements are reflected in motor errors.. J Neurophysiol.

[43] Gritsenko V, Krouchev NI, Kalaska JF (2007). Afferent input, efference copy, signal noise, and biases in perception of joint angle during active versus passive elbow movements.. J Neurophysiol.

[44] Crowe A, Keessen W, Kuus W, van Vliet R, Zegeling A (1987). Proprioceptive accuracy in two dimensions.. Percept Mot Skills.

[45] Slinger RT, Horsley V (1906). Upon the orientation of points in space by the tactile senses of the upper limbs in normal individuals and in blind persons.. Brain.

