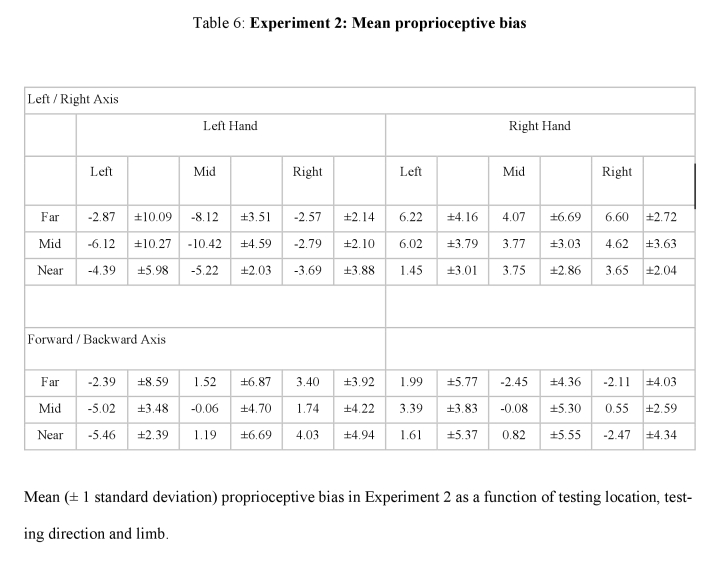# Correction: Mapping Proprioception across a 2D Horizontal Workspace

**DOI:** 10.1371/annotation/5452a5f9-9d97-4be3-a4ca-bca4122b10fc

**Published:** 2010-09-21

**Authors:** Elizabeth T. Wilson, Jeremy Wong, Paul L. Gribble

Tables 3, 4, 5, and 6 contain errors. The +/- sign for all standard deviations was incorrectly converted to a - sign. Please view the correct tables here: 

**Figure:**
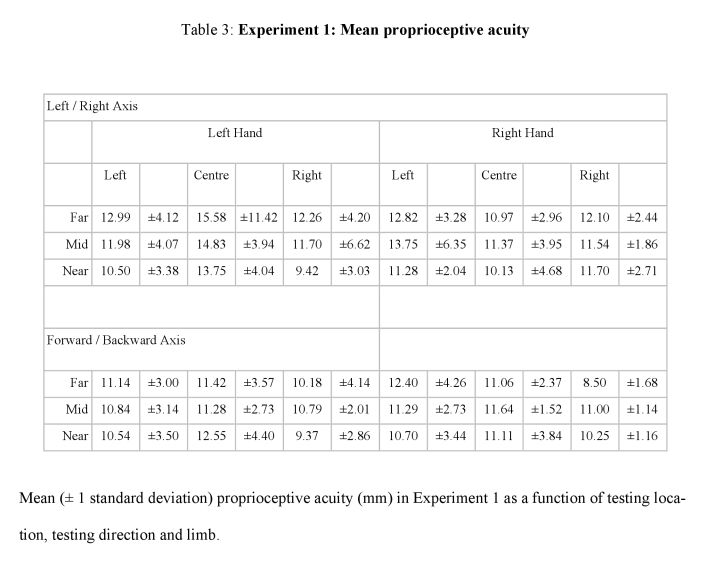


**Figure:**
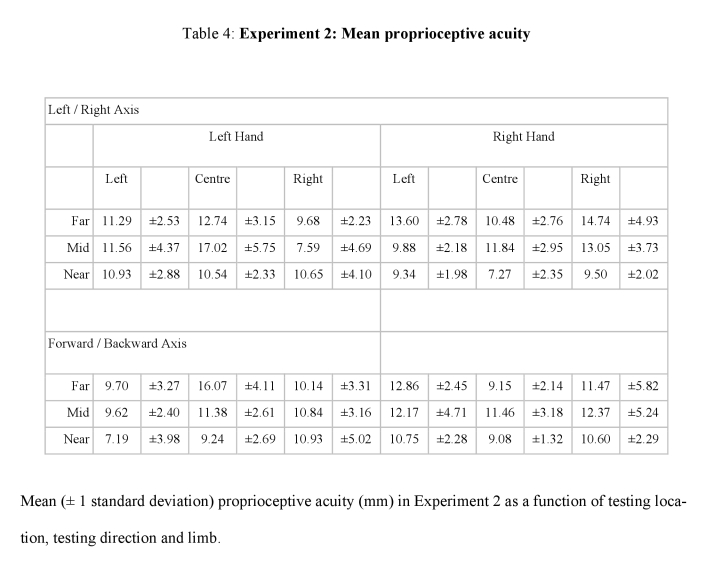


**Figure:**
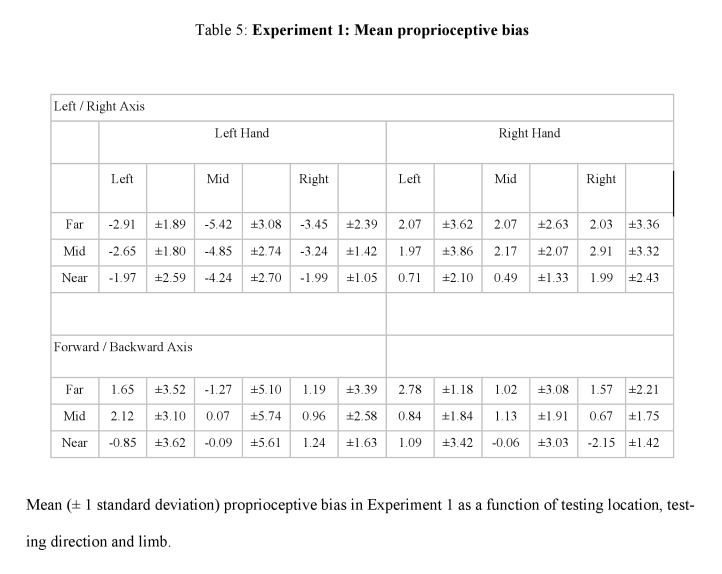


**Figure:**